# Supplementary material for: Cell fate decisions emerge as phages cooperate or compete inside their host
Source: Nat Commun. 2017 Feb 6;8:14341. doi: 10.1038/ncomms14341 (PMC5303824; doi:10.1038/ncomms14341)
Supplement: Supplementary Information — Supplementary Figures, Supplementary Tables, Supplementary Methods and Supplementary References. [file ncomms14341-s1.pdf]

Supplementary Figures

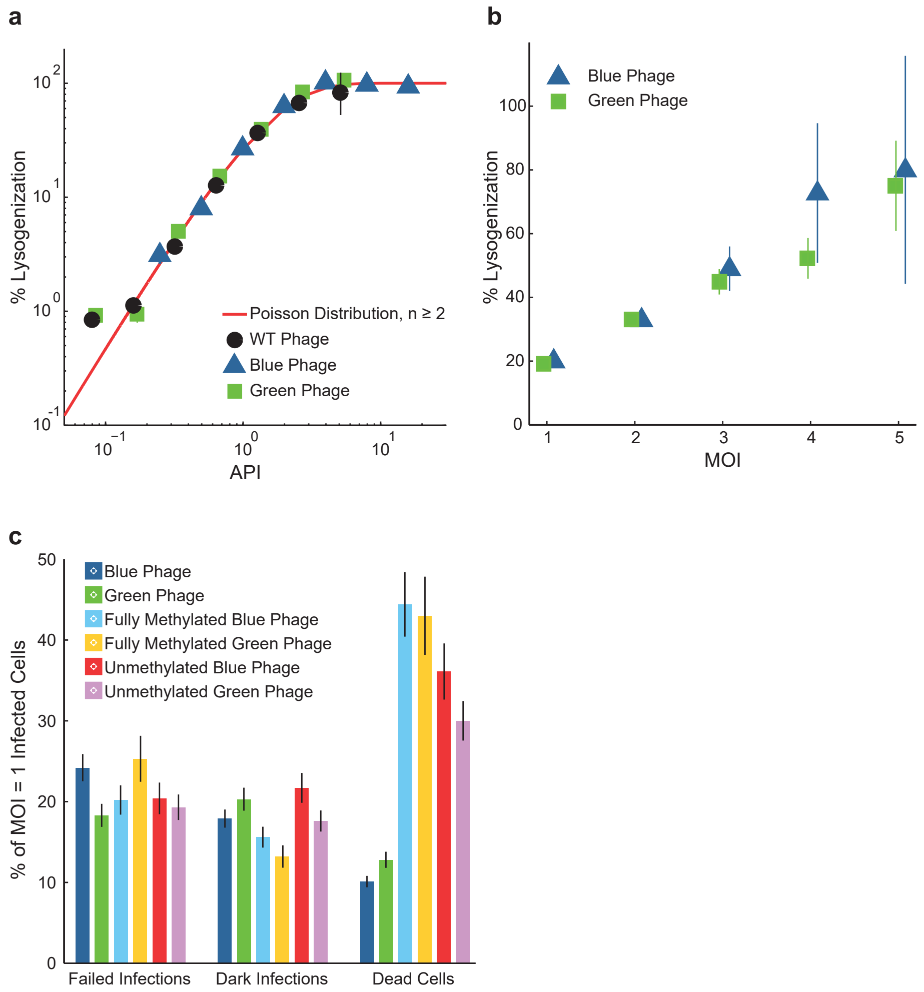

Supplementary Figure 1. Fluorescent phages behave like WT phage.

(a) Fluorescent phages lysogenize like WT phage in bulk. Percentage of lysogeny is plotted against the average phage input (API), showing that fluorescent double reporter phages (blue triangles and green squares represent blue and green phages respectively) are indistinguishable from the wild type (black circles) following the theoretical prediction of Poisson distribution of $n \geq 2$ (red line). Representative plot is shown from 4 biological replicates consisting of 2 technical replicates each. Error bars represent ± s.d. of the technical replicates.

(b) At the single cell level under the microscope, both fluorescent double reporter phages behave similarly to each other, and their lysogenization probabilities increase with multiplicity of infection (MOI). Blue phage data is from movies with N = 179 cells, and green phage data is from movies with N = 200 cells. Error bars represent ± s.e.m.

(c) The failed, dark, and dead cell frequencies for MOI = 1 infections for phages with the normal methylation state (blue and green phages), and fully methylated as well as unmethylated phages. Blue phage data from N = 207 cells, green phage data from N = 164, fully methylated blue phage data from N = 124 cells, fully methylated green phage data from N = 79 cells, unmethylated blue phage data from N = 108 cells, and unmethylated green phage data from N = 150 cells. Error bars represent ± s.e.m.

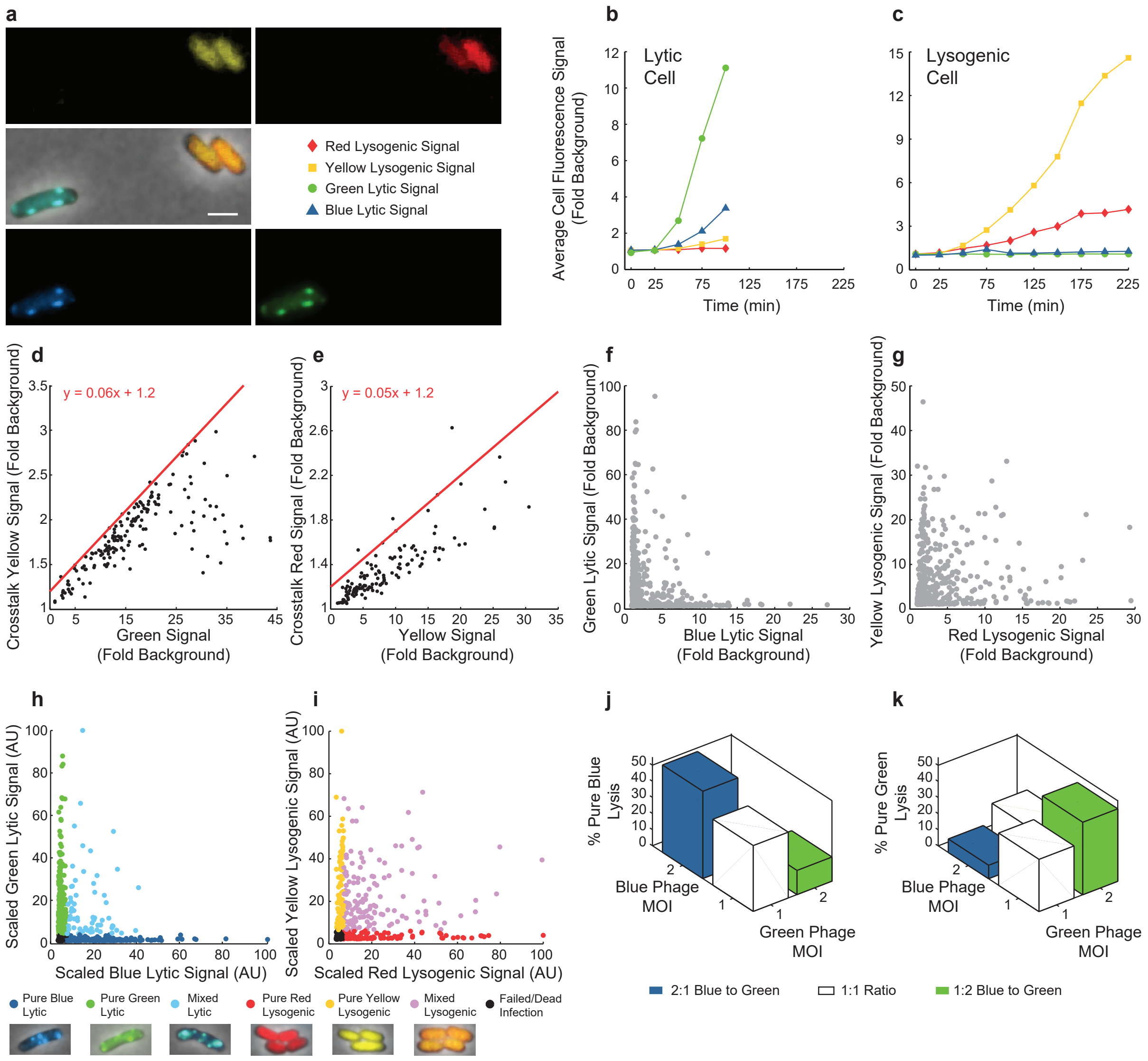

Supplementary Figure 2. Collection and quantification of fluorescent reporter data.

(a) An example of a mixed lytic and mixed lysogenic cell is shown as an overlay image and in each reporter fluorescence channel (blue and green lytic, red and yellow lysogenic).

(b and c) The fluorescence signal in each channel is plotted over time for the example lytic cell (b) and lysogenic cell (c) shown in (a). The signal is defined as the average fluorescence signal within the cell normalized to the average signal of a large cell-free area within the same frame designated as the background.

(d and e) The level of crosstalk of the green into yellow channel (N = 169, green phage) (d) and yellow into red channel (N = 128, blue phage) (e) from pure infections. The crosstalk signals (yellow in d and red in e) are plotted against green and yellow signals from the phages for each cell at the final time point (black dots). The red line shows the upper bound of the crosstalk. The line, which runs above most of the data points, gives a correction factor

where 6% of the green signal is subtracted from the yellow signal (d) and 5% of the yellow signal is subtracted from the red signal (e).

(f and g) The fluorescence signals in the reporter channels at the final time point for each cell are plotted by the lytic (f) and lysogenic (g) channels using the crosstalk corrections, showing a distribution of reporter activities.

(h and i) The highest signal in each channel for lytic/lysogenic cells was scaled to 100 AU and all remaining signals were scaled accordingly. A cutoff value for each channel was assigned to visualize different pure and mixed fates for lytic (h) and lysogenic (i) cells. Representative images of different fates are shown as they appear under the microscope.

(j and k) The percentage of lytic cells showing pure blue (j) and pure green lysis (k) in the total lytic cells infected with specific combinations of phages is plotted against the specific phage MOI on the x and y axes. Scale bars = 2μm.

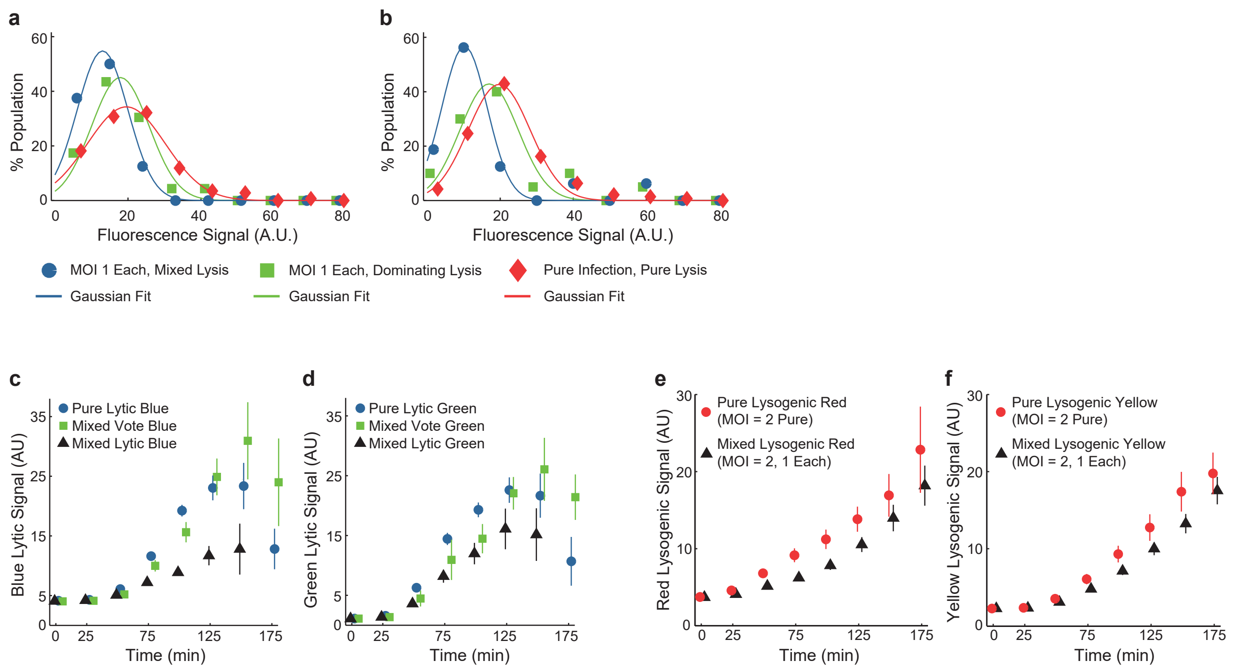

Supplementary Figure 3. Pure and mixed lysis reporter signals behave differently from each other, and from lysogenic signals.

(a and b) Histogram of lytic fluorescence signals before cell lysis and the fitted Gaussian distribution (lines). Lytic cells are grouped into different categories: MOI = 2, one of each phage, mixed lytic (N = 19) (circles, blue and green lytic signals in (a) and (b) respectively); MOI = 2, one of each phage, dominating lytic (squares, blue and green lytic cells in (a) (N = 28) and (b) (N = 24) respectively); pure infections (diamonds, blue and green lytic cells in (a) (N = 168) and (b) (N = 165) respectively).

(c and d) The blue and green lytic signals (c and d respectively) in lytic cells are plotted over time. Lytic cells are divided into groups: pure blue (N = 178) (c) / pure green (N = 179) (d) (circles), mixed lytic (N = 57) blue signal (c) / green signal (d) (triangles), and mixed voting blue lytic (N = 35) (c) / green lytic (N = 20) (d) (squares). Error bars represent ± s.e.m.

(e and f) The yellow and red lysogenic signals (e and f respectively) in lysogenic cells are plotted over time. Lysogenic cells are divided into groups: MOI = 2, pure infections (circles, pure red (N = 11) in (e) and pure yellow (N = 13) in (f), and MOI = 2, one of each phage, mixed lysogens (triangles, N = 35 with red and yellow signals in e and f respectively). Error bars represent ± s.e.m.

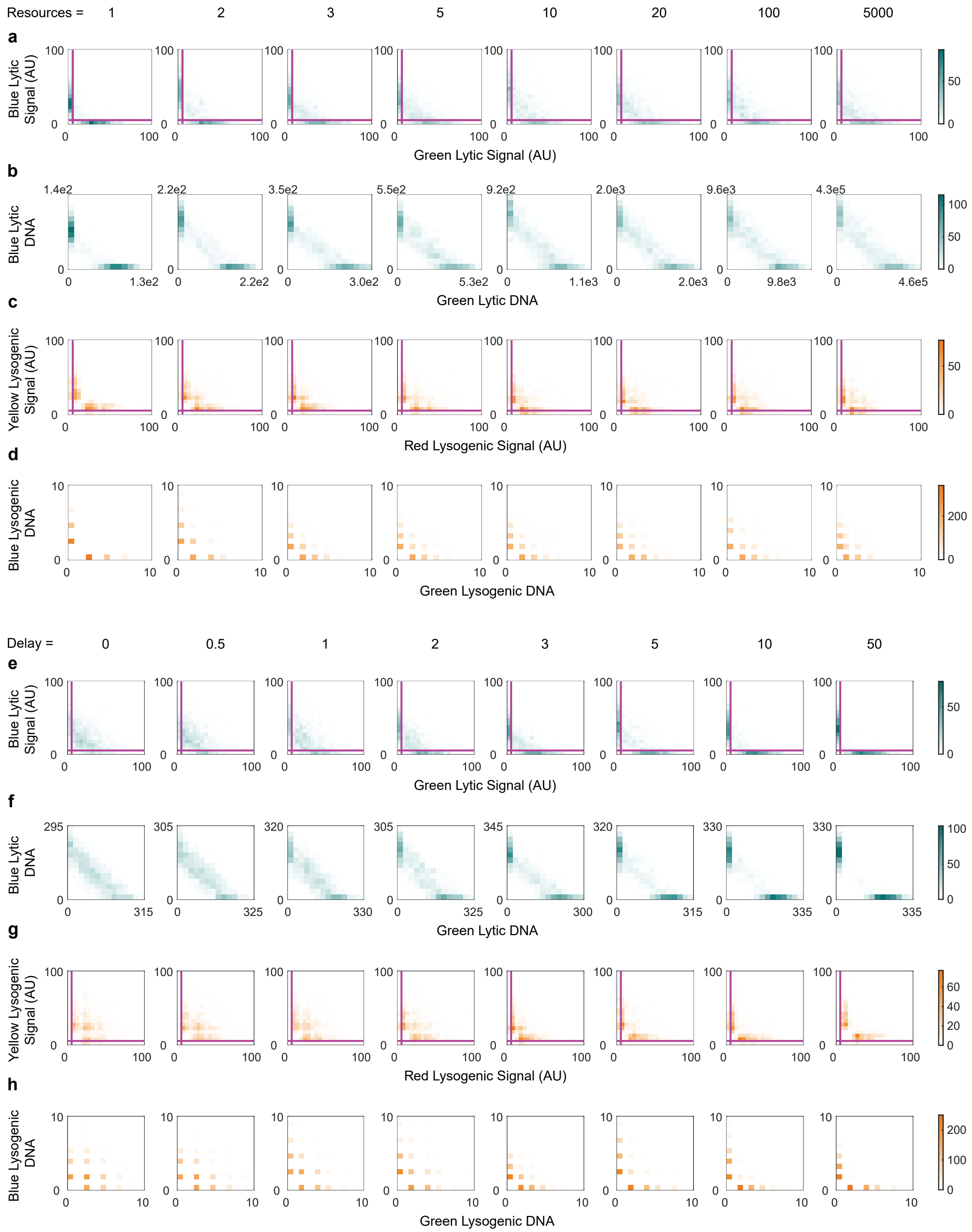
Resources = 1 2 3 5 10 20 100 5000
a
Blue Lytic Signal (AU)
Green Lytic Signal (AU)
b
1.4e2 2.2e2 3.5e2 5.5e2 9.2e2 2.0e3 9.6e3 4.3e5
Blue Lytic DNA
1.3e2 2.2e2 3.0e2 5.3e2 1.1e3 2.0e3 9.8e3 4.6e5
Green Lytic DNA
c
Yellow Lysogenic Signal (AU)
Red Lysogenic Signal (AU)
d
Blue Lysogenic DNA
Green Lysogenic DNA
Delay = 0 0.5 1 2 3 5 10 50
e
Blue Lytic Signal (AU)
Green Lytic Signal (AU)
f
295 305 320 305 345 320 330 330
Blue Lytic DNA
315 325 330 325 300 315 335 335
Green Lytic DNA
g
Yellow Lysogenic Signal (AU)
Red Lysogenic Signal (AU)
h
Blue Lysogenic DNA
Green Lysogenic DNA

Supplementary Figure 4. Resource level and infection timing predicted to imbalance DNA ratio in replication-limited model and influence mixed signal level in lysis, with less effect during lysogeny.
(a-d) The effects of increasing initial resource levels on simulated lytic reporters (a) and DNA numbers (b), and lysogenic reporters (c) and DNA numbers (d) on the simulation end states (N = 1000) using chosen replication-limited parameter set are shown as bivariate histograms. Second phage arrival was fixed at an average of three replications. (e-h) The effects of increasing second phage delays (represented as the number of average replication cycles) on simulated lytic reporters (e) and DNA numbers (f), and lysogenic reporters (g) and DNA numbers (h) on the simulation end states (N = 1000) are shown as bivariate histograms. Resources were fixed at three. Axes of (a), (c), (e), (g) are in units of normalized fluorescence signal; axes of (b), (d), (f), (h) are actual DNA numbers.

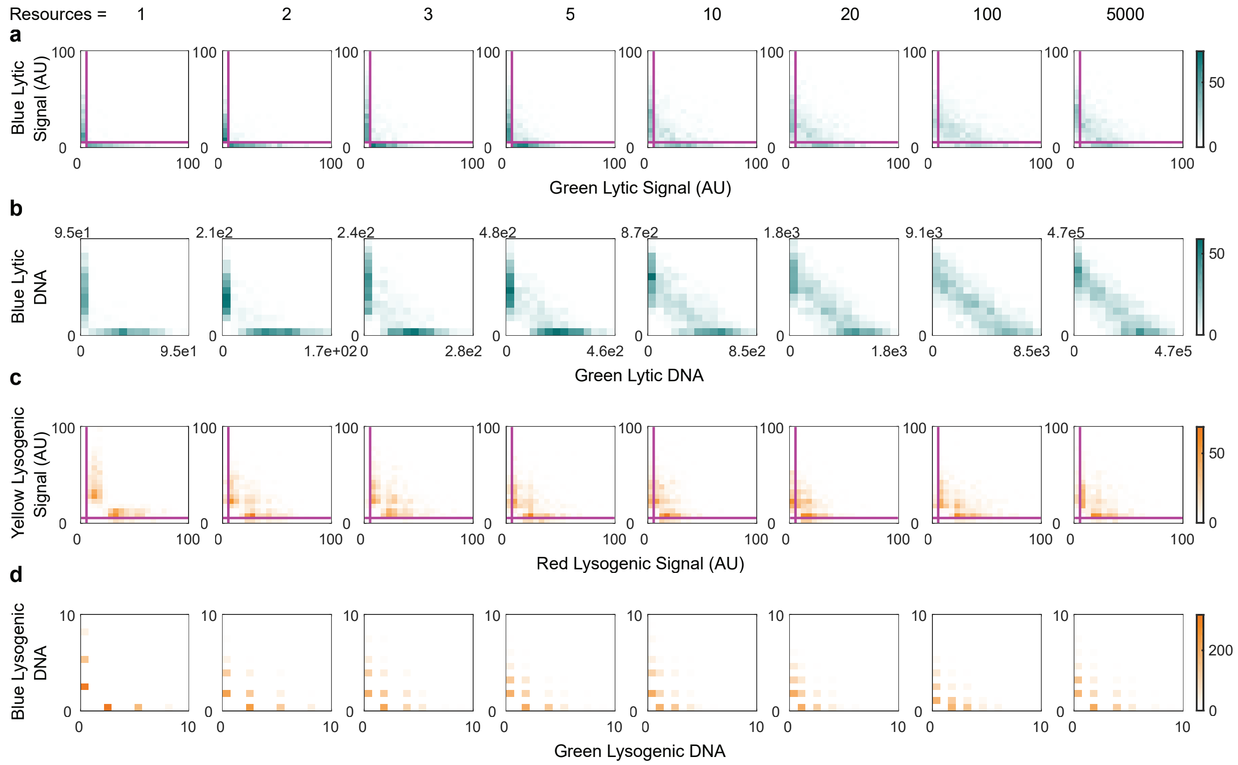

Supplementary Figure 5. Resource level affects DNA and reporter distributions in binding-limited model. (a-d) The effects of increasing initial resource levels on simulated lytic reporters (a) and DNA numbers (b), and lysogenic reporters (c) and DNA numbers (d) on the simulation end states (N = 1000) using binding-limited parameter set are shown as bivariate histograms. Second phage arrival was fixed at an average of two replications. Axes of (a), (c) are in units of normalized fluorescence signal; axes of (b), (d) are actual DNA numbers.

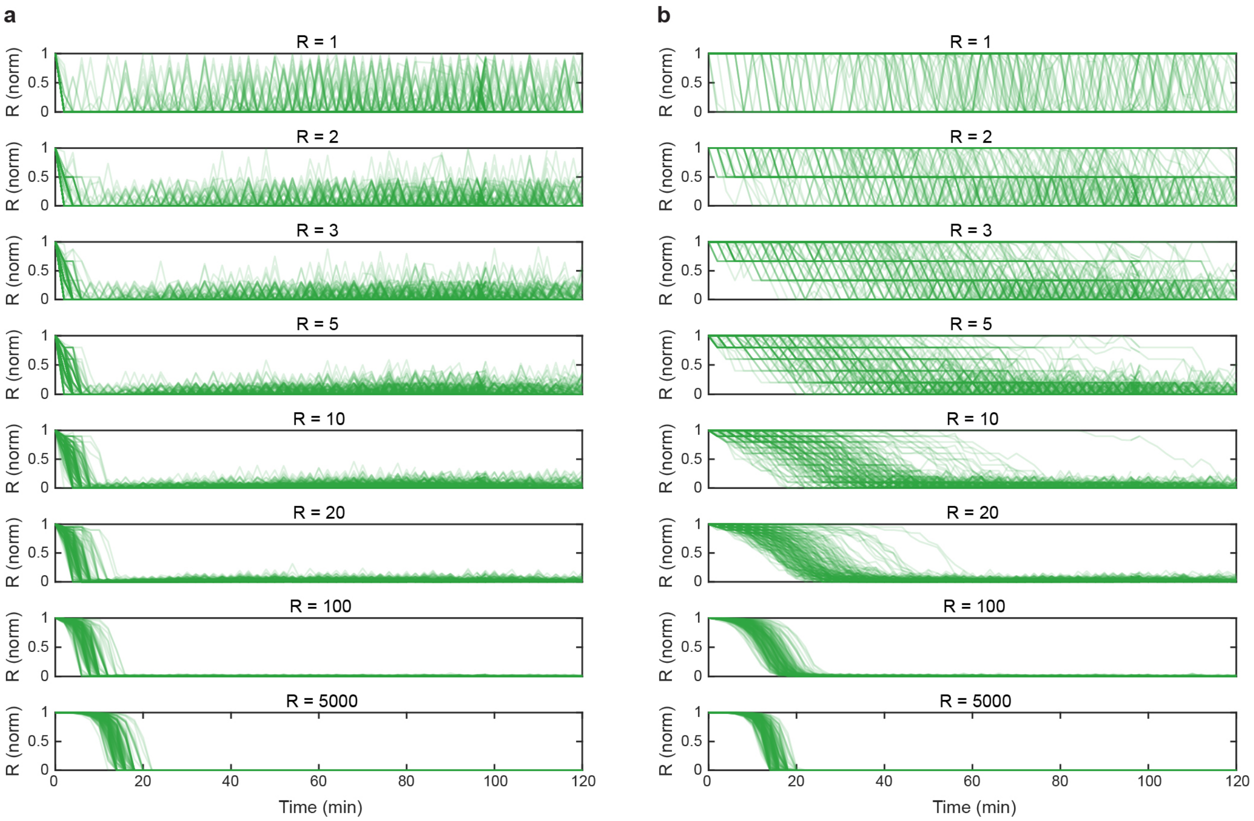

Supplementary Figure 6. Resource usage dynamics changes depending on the model parameters chosen.

Comparison of (a) replication-limited (chosen) with (b) binding-limited parameter sets. The dynamics are more realistic in the replication-limited case, with higher resources being used up later than lower resources.

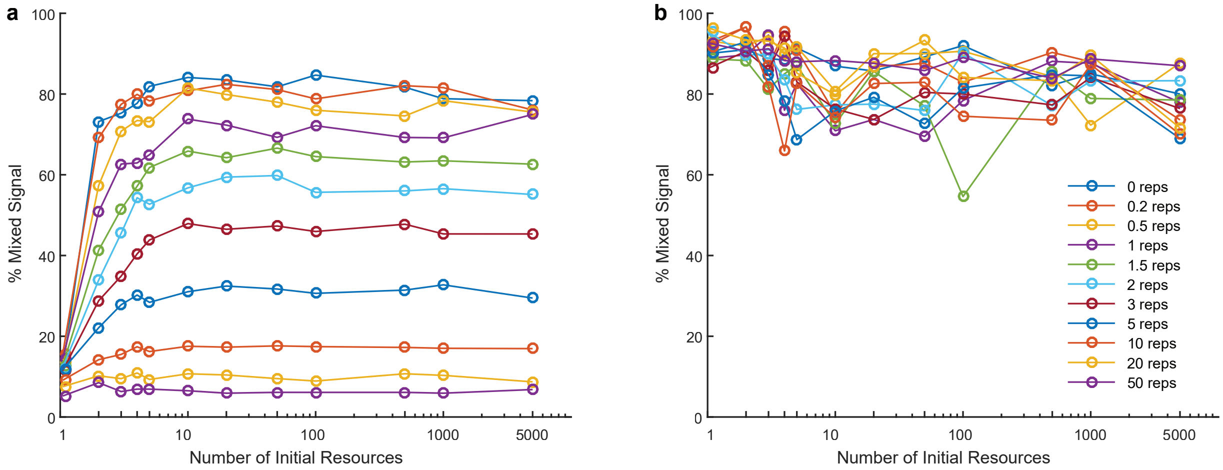

Supplementary Figure 7. Dependence of % mixed lysis and lysogeny on infection timing.

(a) Using our chosen replication-limited model, we simulated different permutations of resource levels and arrival delays on our lytic and lysogenic models. Increasing the arrival (in replication cycles) of the second phage decreases the % mixed lysis for all resource levels. This effect plateaus after around 10-20 replication cycles. (b) Arrival time of the second phage does not have a strong effect on % mixed lysogeny.

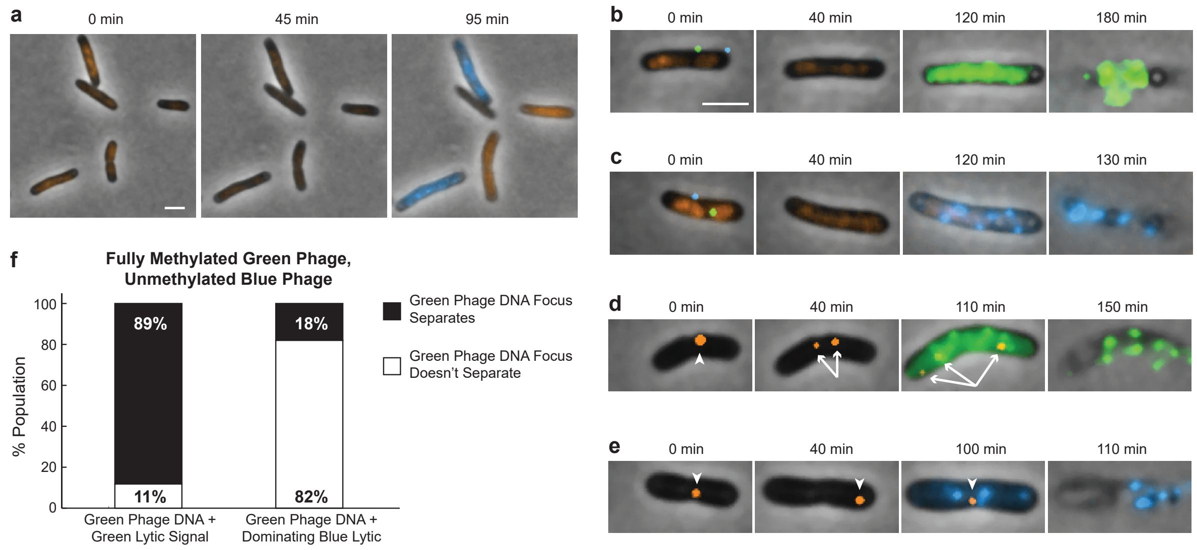

Supplementary Figure 8. DNA reporter cells label green phage DNA and show how DNA is dominated.

(a) Overlay images from an infection movie with unmethylated blue phage infecting the DNA reporter cells are shown over time. No orange foci are observed due to the phage DNA being unmethylated. At 95 min, the fates of the cells are clearly shown to be lytic (blue cells) and lysogenic (orange cells, this cell shares the same fluorescence protein, mKO2 (orange), with the DNA reporter).

(b and c) Overlay fluorescence images of dominating lytic cells due to failed phage DNA ejection for the fully methylated blue (b) and fully methylated green (c) phages (no orange phage DNA foci over time). The cells were lysed by unmethylated green (b) and unmethylated blue (c) phage.

(d and e) The mixed-phage infection of fully methylated green/un-methylated blue phage is similar to that of the reversed methylation state shown in Fig. 5. Arrowheads point to the first appearance of phage DNA (orange dots) and before it divides, and branched arrows point to divided phage DNA. (d) An example of normal lysis: The phage DNA focus is seen at 0 min, and splits into 2 foci at 40 min, then the cell develops green fluorescence and lyses. (e) An example of dominating lysis: The DNA focus (green phage DNA) is present at 0 min, but the DNA focus does not apparently split over time, and the cell lyses with only blue fluorescence.

(f) The lytic cells which have a phage DNA focus (indicating green phage DNA) in mixed-phage infection movies (the green/blue phage is fully/un-methylated) are divided into groups: Lysis with green lytic signal (left, N = 73) and dominating blue lytic (right, N = 17). Within each lytic group, the frequency of the DNA focus separating into

multiple foci is plotted, where the lack of separation is in white and observed separation is in black. As phage DNA replication is required for lysis, 11% of the non-separating group in the population with green lytic signal represents the basal level of failure of the reporter to show phage DNA replication (left). However, 82% (14 of 17 cells) of the non-separating group in dominating blue lytic (right) is much higher than the basal level indicating lack of phage DNA replication could be a reason for this phage (here green phage) being dominated by the blue phage. Of the remaining dominated DNA that does divide, 2/3 showed late ejection into the cell. Scale bars = 2 μm.

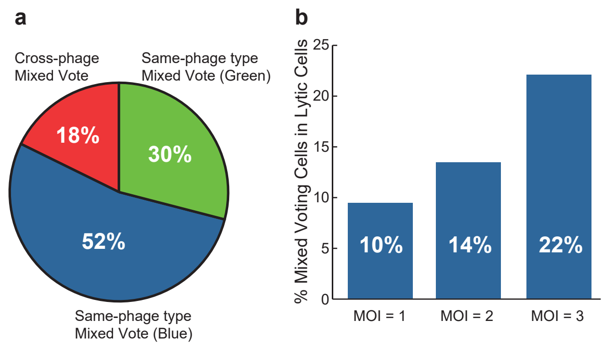

Supplementary Figure 9. Mixed voting cells vary by type and increase with MOI.

(a) Mixed voting cells are grouped into different categories and their frequencies in the mixed voting population are shown (N = 67): cross-phage (lysing with both colors + at least 1 lysogenic color), same-phage type mixed vote blue (blue lytic + yellow lysogenic), and same-phage type mixed vote green (green lytic + red lysogenic).

(b) Mixed voting cells of any category are sorted by their MOI and their frequency is plotted as the percentage population of lytic cells at the given MOIs. The total lytic cells include cells infected with one dark infection. The frequency of mixed voting in lytic cells increases with MOI.

Supplementary Tables

Strain #	Bacterial Strains	Comments	Source
-	MG1655	Wild type *E. coli*	Lab stock
-	LE392	sup^E and sup^F host	Lab stock
LZ1007	MG1655[pBR322-PLate**D*]	Host expressing gpD for titering all λ*D-mNeongreen* phages, Amp^R	This work
LZ1386	MG1655 *seqA-mKO2 Δdam::*Kan^R Cm^R	For methylated phage DNA labeling, Kan^R Cm^R	This work
LZ1387	MG1655 *seqA-mKate2 Δdam::*Kan^R Cm^R	Same as LZ1386, seqA fusion variant, Kan^R Cm^R	This work
LZ1367	MG1655 (λ*D-mTurquoise2* cI_{857}*-mKO2 bor::*Cm^R)[pACYC177-PLate**D*]	Lysogen, induced to produce phage λLZ1367, Cm^R Amp^R	This work
LZ1373	MG1655 (λ*D-mNeongreen* cI_{857}*-mKate2 bor::*Cm^R)[pACYC177-PLate**D*]	Lysogen, induced to produce phage λLZ1373, Cm^R Amp^R	This work
LZ1379	MG1655 (λ*D-mTurquoise2* cI_{857}*-mKO2 bor::*Cm^R)[pACYC177-PLate**D*][pGG503]	Lysogen, induced to produce phage λLZ1379, Cm^R Amp^R Tet^R	This work
LZ1378	MG1655 (λ*D-mNeongreen* cI_{857}*-mKate2 bor::*Cm^R)[pACYC177-PLate**D*][pGG503]	Lysogen, induced to produce phage λLZ1378, Cm^R Amp^R Tet^R	This work
LZ1381	MG1655 dam^- (λ*D-mTurquoise2* cI_{857}*-mKO2 bor::*Cm^R)[pACYC177-PLate**D*]	Lysogen, induced to produce phage λLZ1381, Cm^R Amp^R	This work
LZ1380	MG1655 dam^- (λ*D-mNeongreen* cI_{857}*-mKate2 bor::*Cm^R)[pACYC177-PLate**D*]	Lysogen, induced to produce phage λLZ1380, Cm^R Amp^R	This work
-	LE392[pZA3-*R-Cam-cos*]	Used to make phages with *bor::*Cm^R via recombination, Cm^R Kan^R	Ryland Young
-	LE392[pER157]	Used to make phages with *bor::*Kan^R via recombination, Kan^R Amp^R	Ryland Young
	**Plasmids**		
	pBR322-*D-mTurquoise2-E*	Crossed with phage λLZ1254 to produce phage λLZ1266, Amp^R	This work
	pBR322-*D-mNeongreen-E*	Crossed with phage λLZ1254 to produce phage λLZ1369, Amp^R	This work
	pBR322-cI_{857}*-mKO2-partRexB*	Crossed with phage λcI^- to produce phage λLZ1357, Amp^R	This work
	pBR322-cI_{857}*-mKate2-partRexB*	Crossed with phage λcI^- to produce phage λLZ1355, Amp^R	This work
	pBR322-PLate**D*	Expresses wild type gpD for phage stability, Amp^R	This work
	pACYC177-PLate**D*	Expresses wild type gpD for phage stability, lower expression than pBR322-PLate*D , Amp^R	This work
	pZA3-*R-Cam-cos*	Plasmid for recombination, *bor::*Cm^R	Ryland Young
	pER157	Plasmid for recombination, *bor::*Kan^R	Ryland Young
	pKD46	Arabinose-induced λ red recombination, Amp^R	Ryland Young

Supplementary Table 1. Bacterial strains and plasmids used in this work.

Phage #	Phage Strains	Comments	
λLZ613	λcI_{857} *bor::*Kan^R	"λWT-*Kan*," wild type phage, Kan^R	Lab Stock
λLZ859	λcI_{857} *bor::*Cm^R	"λWT-*Cm*," wild type phage, Cm^R	Lab Stock
λLZ637 or λP^-	λcI_{857} Pam_{80} *bor::*Kan^R	"λP^--*Kan*," phage with nonsense mutation in *P*, Kan^R	Lab Stock
λLZ895	λcI_{857} cII_{68} *bor::*Kan^R	"λcII^--*Kan*," phage with point mutation in *cII*, Kan^R	Lab Stock
λLZ896	λcI_{857} cII_{68} *bor::*Cm^R	"λcII^--*Cm*," phage with point mutation in *cII*, Kan^R	Lab Stock
λLZ1367	λ*D-mTurquoise2* cI_{857}*-mKO2 bor::*Cm^R	"Blue phage," double reporter, Cm^R	This work
λLZ1373	λ*D-mNeongreen* cI_{857}*-mKate2 bor::*Cm^R	"Green phage," double reporter, Cm^R	This work
λLZ1381	Unmethylated λ*D-mTurquoise2* cI_{857}*-mKO2 bor::*Cm^R	"Unmethylated blue phage," Cm^R	This work
λLZ1380	Unmethylated λ*D-mNeongreen* cI_{857}*-mKate2 bor::*Cm^R	"Unmethylated green phage," Cm^R	This work
λLZ1379	Fully methylated λ*D-mTurquoise2* cI_{857}*-mKO2 bor::*Cm^R	"Fully methylated blue phage," Cm^R	This work
λLZ1378	Fully methylated λ*D-mNeongreen* cI_{857}*-mKate2 bor::*Cm^R	"Fully methylated green phage," Cm^R	This work
-	λ*Dam* cI_{857}	Phage with nonsense mutation in *D*	Alan Davidson
λLZ1254	λ*Dam* cI_{857} *bor::*Kan^R	Phage with nonsense mutation in *D,* used in recombination to produce phages λLZ1266 & λLZ1369, Kan^R	This work
λLZ1266	λ*D-mTurquoise2* cI_{857} *bor::*Kan^R	Crossed with phage λLZ1357 to make LZ1367, Kan^R	This work
λLZ1369	λ*D-mNeongreen2* cI_{857} *bor::*Kan^R	Crossed with phage λLZ1355 to make LZ1373, Kan^R	This work
-	λcI^-	λ*cI* point mutant, used in recombination to produce phages λLZ1357 & λLZ1355	Ryland Young
λLZ1357	λcI_{857}*-mKO2 bor::*Cm^R	Crossed with phage λLZ1266 to make LZ1367, Cm^R	This work
λLZ1355	λcI_{857}*-mKate2 bor::*Cm^R	Crossed with phage λLZ1369 to make LZ1373, Cm^R	This work

Supplementary Table 2. Bacteriophages used in this work.

Parameter	Value	Units	Meaning	Reasoning/reference
k_1	1	s^{-1}	Phage DNA binds DNA pol/replisome	Fast diffusion of proteins[1]
k_2	1e-2	s^{-1}	New DNA production by complex	Of the order of minutes[2]
k_3	1e-3	s^{-1}	Replisome unbinds from DNA	Infrequent because replisomes thought to stay bound[3]
k_4	5e-2	s^{-1}	Lytic reporter protein production	Gives reasonable lytic reporter numbers, of gpD[4]
k'_1	1	s^{-1}	Lysogenic DNA (unreplicated and replicated) binds DNA pol/replisome	As k_1
k'_2	1e-2	s^{-1}	New DNA production by complexes (unreplicated and replicated)	As k_2
k'_3	1e-3	s^{-1}	Replisome unbinds from lysogenic DNA	As k_3
k'_4	2e-3	s^{-1}	Lysogenic reporter production	Gives reasonable lysogenic reporter numbers[5]. Slower than k_4 as following initial spike of CI production, cI regulates itself to slower stable production[6]
k'_5	2e-2	s^{-1}	Replicated DNA/complex turns into lysogen	Tuned to resemble experimental data, expected to be on the order of DNA replication as lysogeny typically requires replication[7]
k'_6	1e0	s^{-1}	Forced lysogenization by lysogens	Fast diffusion of CI[1]

Supplementary Table 3. Parameters used in the computational models.

Supplementary Methods

Reporter phage strain construction

First, λ*D-mTurquoise2/mNeongreen* cI_{857} *bor::Kan*^{R} phages were produced by infecting λ*Dam* cI_{857} *bor::Kan*^{R} phages on LE392 (permissive host) bearing plasmids pBR322-λ*D-mTurquoise2/mNeongreen-E* for recombination. The resulting lysate contained the phage of interest, and was titered on a non-permissive MG1655 to select for recombinants, and the resulting plaques were screened with a fluorescence dissecting microscope to pick fluorescent plaques, yielding λ*D-mTurquoise2/mNeongreen* cI_{857} *bor::Kan*^{R} phages which were amplified and lysogenized into MG1655. Separately, λcI_{857}*-mKO2/mKate2 bor::Cm*^{R} phages were produced by infecting λcI-phages onto MG1655 bearing plasmids with λcI_{857}*-mKO2/mKate2* for recombination at 42 ^{o}C to ensure that the temperature-sensitive[8] CI_{857} from the plasmid was inactivated. The resulting lysate contained the phage of interest and was titered on MG1655 at 30 ^{o}C, a temperature permissive for lysogenization, and recombinant phages were screened by picking turbid plaques, yielding λcI_{857}*-mKO2/mKate2* phages. The phages were then crossed with plasmid pZA3-*R-Cam-cos* (gift of Ryland Young) to replace bor with the antibiotic resistance cassette through recombination[9], yielding λcI_{857}*-mKO2/mKate2 bor::Cm*^{R} phages. Then co-infection of pairs of phages, λ*D-mTurquoise2* cI_{857} *bor::Kan*^{R}/λcI_{857}*-mKO2 bor::Cm*^{R} and λ*D-mNeongreen* cI_{857} *bor::Kan*^{R}/λcI_{857}*-mKate2 bor::Cm*^{R}, was done for recombination to generate the final strains for the experiments, λ*D-mTurquoise2* cI_{857}*-mKO2 bor::Cm*^{R} and λ*D-mNeongreen* cI_{857}*-mKate2 bor::Cm*^{R}. Screening was done by titering the lysate from the crossing infection at 30 ^{o}C to produce turbid plaques, and then small turbid plaques, which are likely fluorescent phages, were selected for Cm resistance, indicating a cross had occurred. The strains were confirmed through PCR and microscopy to be single integration lysogens with the correct fluorescence combinations. The lysogens were transformed with the plasmid pACYC177-PLate**D* to produce mosaic WT and fluorescent gpD progeny phages upon induction. Phages with λ*D-mNeongreen* were titered on MG1655[pBR322-PLate**D*] to generate stable phages to make plaques.

The phage lysogens were then transformed with the pACYC177-PLate**D* plasmid in order to generate stable fluorescent phages. The unmethylated version of these phages were produced by infecting *dam*$^-$ cells[10] with these phages and then transforming those verified single integration lysogens with the pACYC177-PLate**D* plasmid (generating LZ1380 and LZ1381). The fully methylated version of these phages was produced by transforming pGG503 plasmid[11] into the lysogens already bearing pACYC177-PLate**D* plasmid (transformed LZ1367 and LZ1373 to generate LZ1379 and LZ1378 respectively), followed by heat induction of the lysogens.

Phage purification by ultracentrifugation

The purification steps were as described[12, 13]. Briefly, a single colony of the lysogens of the desired phages was first grown in 5 ml of LB+10 mM $MgSO_4$ (LBM) with appropriate antibiotics at 30 ^oC overnight. The overnight culture was then diluted 1:100 into 500 ml of fresh LBM with appropriate antibiotics and grown at 30 ^oC until OD_{600} ~ 0.4, then moved to 42 ^oC for 20 min, then moved to 37 ^oC until lysis occurred. 2% volume of chloroform was then added, and left to shake gently on an orbital shaker at room temperature for 15 min, then the lysate excluding the chloroform was centrifuged at 10,000 x g for 20 min, the resulting supernatant was then centrifuged again under the same conditions. The new supernatant was then treated with DNase I (Sigma) and RNase A (Sigma) with a concentration of 1 μg/ml each at room temperature for 1 hour, and then NaCl was added to reach a concentration of 1 M, and incubated on ice for ~3 hours. The mixture was then centrifuged as before and PEG8000 (Fisher Scientific) was added to reach 10% w/v, and the mixture was left at 4 ^oC with gentle shaking overnight, ~16 hours. The next day, the mixture was centrifuged at 3000 x g and the supernatant was discarded. The pellet was soaked in a total of 8 ml of cold SM buffer and incubated at 4 ^oC overnight, ~16 hours. The resuspended pellet was removed and the centrifugation bottles were rinsed with 1-2 ml of SM buffer and combined, then the SM suspension was mixed gently with an equal volume of chloroform and centrifuged at ~3700 x g for 15 min at 4 ^oC. The supernatant was removed to exclude the PEG pellet, and the process starting from the chloroform mixing was done 2 more times, to finally yield a clear supernatant containing the phage. A step gradient was made for each desired phage using 1.5 ml each of 1.3, 1.5, and 1.7 g/ml CsCl (Sigma) +SM buffer solutions, and the phage (~ 8 ml) was layered on top in a 13.2 ml ultra-clear tube (Beckman Coulter), then ultracentrifuged in a Beckman SW 41Ti rotor at 24,000 rpm for 6-8 hours at 4 ^oC . The phage migrates to a band and is then extracted using a 3 ml syringe (Becton Dickinson) and 20 gauge needle (Becton Dickinson) from the side wall of the tube. This phage extraction was then loaded a 5 ml ultra-clear tube (Beckman Coulter) and then filled by a 1.5 g/ml CsCl+SM buffer and ultracentrifuged in a Beckman SW 50 rotor at 35,000 rpm for 24 hours at 4 ^oC, and then was extracted in the same manner. This new phage extraction was then loaded into a dialysis cassette and dialyzed 1:1000 against SM buffer in three steps for a total of ~24 hours. This phage solution was then extracted and stored away from light and at 4 ^oC to be used in the experiments. DAPI (Sigma) staining was done to verify that the phages have DNA.

Frequencies of failed, dark and dead infection

For failed infection frequencies, those cells observed to be infected with a single blue/green phage were selected from the mixed-phage infection movies (MOI = 1 infections). If a cell showed no fluorescence development and grew and divided over the movie, it was marked as a failed infection. The failed infection frequency is the number of failed infections over the total number of MOI = 1 infections for the respective phage.

For dark infection frequencies, those cells with no phage attached in the first frame were selected from the mixed-phage infection movies (MOI = 0 infections). If a cell showed a pure fate (pure blue/green lysis or pure red/yellow lysogenic) or same-phage mixed voting (blue with yellow or green with red development), it was marked as a dark infection. The dark infection frequency is the number of dark infections over the total number of MOI = 1 infections plus the number of dark infections.

For dead cell infection frequencies, those cells observed to be infected with a single blue/green phage were selected from the mixed-phage infection movies (MOI = 1 infections). If a cell did not grow and divide over the movie (regardless of lysogenic fluorescence), lysed without lytic fluorescence, or filamented without any fluorescence, it was marked as a dead cell infection. The dead cell infection frequency is the number of dead cell infections over the total number of MOI = 1 infections for the respective phage.

The frequencies were shown in Supplementary Fig. 1c.

Calculation of pure cell fate due to failed and dark infections.

Assume that the frequencies of phage failed and dark infections for MOI = 1 are *a* and *b* respectively, and the frequencies of failed or dark infection for one infected cell are a^n or b^n, where n is the number of failed or dark phages. Here we only consider n = 1 as the frequencies are low when n > 1. The failed and dark probabilities we use for our calculations, a and b, are the failed and dark infections frequencies of the blue and green phages averaged together (data from Supplementary Fig. 1c). From our measurements, we found a = 21.5±1.1% and b =19.1±0.9%, where the errors are the propagated s.e.m. from the measured failed and dark infection frequencies.

For mixed MOI = 2, one of each phage, the probabilities of pure infection of blue phage (p_1), pure infection of green phage (p_2), dual-color infection (p_3), and both phage failed infection (p_4) are:

$$p_1 = (1-a)\times a\times\left(1-b+\frac{b}{2}\right)+b\times b\times\frac{b}{2} = 0.157$$

$$p_2 = p1 = 0.157$$

$$p_4 = a\times a\times(1-b) = 0.037$$

$$p_3 = 1 - p_1 - p_2 - p_4 = 0.649$$

As we ignored the cases of the both phage failed infection in the experiments, p1, p2, and p3 are then adjusted to:

The probability of pure infection of phage 1 (f_1): $f_1 = p_1/(1 - p_4) = 0.163$

The probability of pure infection of phage 2 (f_2): $f_2 = p_2/(1 - p_4) = 0.163$

Therefore, the probability of pure infection is $f_1 + f_2 = 0.326$

The probability of dual-color infection (f_3): $f_3 = p_3/(1 - p_4) = 1 - f_1 - f_2 = 0.674$

For mixed MOI = 3, similarly,

$$p_1 = (1 - a^2)\times a\times \left(1 - b + \frac{b}{2}\right) + a\times a\times a\times \frac{b}{2} = 0.186$$

$$p_2 = a^2\times(1 - a)\times \left(1 - b + \frac{b}{2}\right) + a\times a\times a\times \frac{b}{2} = 0.034$$

$$p_4 = a\times a\times a\times(1 - b) = 0.008$$

The probability of pure infection of phage 1 (f_1): $f_1 = p_1/(1 - p_4) = 0.188$

The probability of pure infection of phage 2 (f_2): $f_2 = p_2/(1 - p_4) = 0.034$

Therefore, the probability of pure infection is $f_1 + f_2 = 0.222$

The probability of dual-color infection (f_3): $f_3 = 1 - f_1 - f_2 = 0.778$

For MOI = 4, similarly,

$$p_1 = (1 - a^2)\times a\times a\times(1 - b + b/2) + a\times a\times a\times a\times b/2 = 0.040$$

$$p_2 = p_1 = 0.040$$

$$p_4 = a\times a\times a\times a\times(1 - b) = 0.002$$

The probability of pure infection of phage 1 (f_1): $f_1 = p_1/(1 - p_4) = 0.040$

The probability of pure infection of phage 2 (f_2): $f_2 = p_2/(1 - p_4) = 0.040$

Therefore, the probability of pure infection is $f_1 + f_2 = 0.080$

The probability of dual-color infection (f_3): $f_3 = 1 - f_1 - f_2 = 0.920$

Computational Methods

We formulated two separate simple biochemical reaction models, one for the lytic fate and one for the lysogenic one. In both, we assumed that the starting point was upon entry of the first of two phage DNAs into the host cell. The biochemicals involved in the models are viral DNA (lytic DNA *V*, unreplicated pre-lysogenic DNA *N*, replicated pre-lysogenic DNA *P*, and lysogens *L*), an unspecified resource that we assumed to be host

replisomes/replisome component (*R*), DNA-replisome complexes (C_V, C_N, and C_P), and fluorescent reporters (*D* and *E*).

The goal of the models was to examine the interactions between two individual phages and the resource, so there were two sets of each biochemical species: one to represent the blue and one for the green phage, and copies of their DNA and proteins. The models consisted of the reactions:

Lytic:

1. $V_1 + R \xrightarrow{k1} C_{V1}$ — Viral replication: phage DNA binds DNA pol/replisome
2. $V_2 + R \xrightarrow{k1} C_{V2}$
3. $C_{V1} \xrightarrow{k2} V_1 + C_{V1}$ — Viral replication: DNA production
4. $C_{V2} \xrightarrow{k2} V_2 + \mathrm{C}_{\mathrm{V2}}$
5. $C_{V1} \xrightarrow{k3} V_1 + R$ — Replisome unbinds from DNA
6. $C_{V2} \xrightarrow{k3} V_2 + R$
7. $V_1 \xrightarrow{k4} V_1 + D_1$ — Lytic reporter protein production
8. $V_2 \xrightarrow{k4} V_2 + D_2$

Lysogenic:

1. $N_1 + R \xrightarrow{k\prime 1} C_{N1}$ — Viral replication: unreplicated DNA binds DNA pol/replisome
2. $N_2 + R \xrightarrow{k\prime 1} C_{N2}$
3. $P_1 + R \xrightarrow{k\prime 1} C_{P1}$ — Viral replication: replicated DNA binds DNA pol/replisome
4. $P_2 + R \xrightarrow{k\prime 1} C_{P2}$
5. $C_{N1} \xrightarrow{k\prime 2} C_{P1} + P_1$ — Viral replication: unreplicated DNA becoming replicated DNA/complex
6. $C_{N2} \xrightarrow{k\prime 2} C_{P2} + P_2$
7. $C_{P1} \xrightarrow{k\prime 2} C_{P1} + P_1$ — Viral replication: replicated DNA complex producing replicated DNA
8. $C_{P2} \xrightarrow{k\prime 2} C_{P2} + P_2$
9. $C_{N1} \xrightarrow{k\prime 3} N_1 + R$ — Replisome unbinds unreplicated DNA
10. $C_{N2} \xrightarrow{k\prime 3} N_2 + R$
11. $C_{P1} \xrightarrow{k\prime 3} P_1 + R$ — Replisome unbinds replicated DNA
12. $C_{P2} \xrightarrow{k\prime 3} P_2 + R$
13. $L_1 \xrightarrow{k\prime 4} L_1 + E_1$ — Lysogenic reporter production
14. $L_2 \xrightarrow{k\prime 4} L_2 + E_2$
15. $P_1 \xrightarrow{k\prime 5} L_1$ — Replicated DNA switch to lysogen

16. $P_2 \xrightarrow{k'5} L_2$

17. $C_{P1} \xrightarrow{k'5} L_1$ Replicated DNA complex switch to lysogen

18. $C_{P2} \xrightarrow{k'5} L_2$

19. $N_1 + L_2 \xrightarrow{k'6} L_1 + L_2$ Forced lysogenization of naive DNA by lysogen

20. $N_2 + L_1 \xrightarrow{k'6} L_2 + L_1$

21. $C_{N1} + L_2 \xrightarrow{k'6} L_1 + L_2$ Forced lysogenization of unreplicated complex DNA by lysogen

22. $C_{N2} + L_1 \xrightarrow{k'6} L_2 + L_1$

23. $P_1 + L_2 \xrightarrow{k'6} L_1 + L_2$ Forced lysogenization of replicated DNA by lysogen

24. $P_2 + L_1 \xrightarrow{k'6} L_2 + L_1$

25. $P_1 + L_1 \xrightarrow{k'6} 2L_1$

26. $P_2 + L_2 \xrightarrow{k'6} 2L_2$

27. $C_{P1} + L_2 \xrightarrow{k'6} L_1 + L_2$ Forced lysogenization of replicated DNA complex by lysogen

28. $C_{P2} + L_1 \xrightarrow{k'6} L_2 + L_1$

29. $C_{P1} + L_1 \xrightarrow{k'6} 2L_1$

30. $C_{P2} + L_2 \xrightarrow{k'6} 2L_2$

The two main premises of the lytic model are that the phage DNA replicates, and that in order to replicate it must temporarily use up a resource, which it releases some time later. The resource may be a host replisome or replisome component such as polymerase, which binds to DNA and allows it to replicate[14]. The resource is kept bound after replication, as DNA replication complexes are thought to be inherited following replication[15]. We hypothesized that these replisomes were the limiting factor in the phage competition, since it is known that there are very few of them in an *E. coli* cell[16, 17].

The lysogenic model is simply an expanded model of the lytic one, with the difference that DNA initially starts off as 'naïve' DNA, which must then convert into lysogen by integrating into the host genome. We added a further layer of complexity by forcing the naïve DNA to replicate at least once before becoming a lysogen, as phage lambda requires DNA replication to efficiently lysogenize[7]. In addition, we allowed lysogens to force naïve and replicated DNA to themselves turn into lysogens, to account for the action of CI protein[6].

These lytic and lysogenic models were both simulated using the tau-leap[18] stochastic simulation method developed by Daniel T. Gillespie as an offspring of his original stochastic simulation algorithm[19]. This is an approximate but fast simulation method that samples the biochemical system at various time points (the frequency of which can be influenced by an error parameter, which controls the trade-off between accuracy and speed; we set

this as 0.1). It is ideal for quickly simulating biochemical systems that may have reactions with disparate propensities (frequent and rare reactions). Such stochastic methods take into account the randomness of biochemical reactions within the cell, which can become important at low copy numbers, e.g. of genes.

These models assume an MOI = 2 and the key parameters that are varied are the second phage arrival delay and the initial resource level. The simulation also ends after a variable time, with the exact end time for each simulation being chosen from a normal distribution. For the lytic simulations, we ran the simulation for 6,600 ± 1200 seconds, which is roughly the average lysis time and standard deviation of the experimental data for normal lytic cells (Fig. 6c). The lysogenic model ends after 13,500 ± 60 seconds, which roughly corresponds to the time point that the experimental lysogenic data was taken, and the standard deviation is based on the fact that the images at different stages for each time point are taken up to a few minutes apart due the automatic image capturing.
We ran 1000 simulations for each set of conditions. We simulated these systems of equations for resource levels of 1, 2, 3, 5, 10, 50, 100, 500, 1000, and 5000. These models assume an MOI = 2. In each of simulation, we also varied the mean arrival delay of the second phage, defined in units of number of replication cycles (1 cycle = 100 seconds), in order to more intuitively link it to biological considerations. The values we tested were 0, 0.2, 0.5, 1, 1.5, 2, 3, 5, 10, 20, and 50 replication cycles. When we tested the effect of varying the arrival delays or resource levels we set the other variable to that of our representative parameter set. The actual arrival delay for both phages in each simulation was drawn from an exponential distribution with a mean of the arrival delay we chose. These arrival times were both then normalized so the first phage arrived at $t = 0$. In addition, the simulation runtimes were also stochastic, sampled from a normal distribution with the same mean and standard deviation as our experimental data. In the rare cases that the arrival time of the second phage exceeded the simulation time, the runtime was re-sampled.

Starting out with the lytic model, we performed a brief parameter scan to find the optimal regime that best resembled the experimental data. We found two sets of parameters that both had reasonable behaviour, in terms of the % pure and mixed reporter signals and the actual numbers of phage DNA, lysogens and reporters. The first one, which we call replication-limited, had $k_1 > k_2$, and vice versa for the second one, which we call binding-limited. The main difference between the two was usage of the resource: in the replication-limited schema, binding of phage DNA to resource was fast, and so all DNA quickly formed complexes that began to replicate. In the binding-limited schema, binding was much slower; because of this there was a reversal of the expected order, and in simulations with high resources, they were bound faster than in those with low resources. We chose to work with the replication-

limited schema, as this was more biologically realistic in terms of both the likely reaction rates of the processes, as well as the resource usage dynamics. As constraints, we were able to use lytic DNA, lysogen and reporter numbers, which are roughly known in the literature (Supplementary Table 3). Once we had chosen the parameters for the lytic model, we based the lysogenic model on these and set $k'_1 = k_1$, $k'_2 = k_2$, and $k'_3 = k_3$. However, we chose a different reporter protein production rate in order for the model to match known stable numbers of CI protein, and it is known that CI regulates itself to a low level with slower production[6]. To find k'_5 and k'_6, we ran another parameter scan, picked the parameter region that most resembled the data, then manually fine-tuned these parameters.

We normalized the fluorescent reporter signals, in order to compare to experimental results, as:

$$\text{normalized data} = \frac{\text{data} - \min(\text{data})}{\max(\text{data}) - \min(\text{data})}$$

To create bivariate histograms, these normalized data were then binned into bins of width 5%, in both their blue and green lytic fluorescence signals (or red and yellow lysogenic signals). Percent mixed signals were calculated by setting a threshold of 5%; if both lytic/lysogenic fluorescence signals were above this value the simulated cell was labeled as mixed; of one signal was > 5% but the other was < 5%, it was labelled as pure. When both signals were < 5%, those results were labelled as dead cells and discard. The frequency of these was < 0.3% for all conditions. From here, the number of pure and mixed infections divided by the total simulation number yielded the prevalence of each infection fate.

For our representative simulations (n = 71 and n = 49 for lytic and lysogenic, respectively), we used a resource level of 3 and average delay of 3 cycles (about 5 minutes), as under these conditions, the simulations resemble our experimental data, and are biologically realistic[20, 21, 22].

Supplementary References

1. Elowitz MB, Surette MG, Wolf PE, Stock JB, Leibler S. Protein mobility in the cytoplasm of Escherichia coli. *Journal of bacteriology* **181**, 197-203 (1999).

2. Better M, Freifelder D. Studies on the replication of E. coli phage lambda DNA. *Virology* **126**, 168-182 (1983).

3. Wegrzyn G, Taylor K. Inheritance of the replication complex by one of two daughter copies during lambda plasmid replication in Escherichia coli. *J Mol Biol* **226**, 681-688 (1992).

4. Casjens SR, Hendrix RW. Locations and amounts of major structural proteins in bacteriophage lambda. *J Mol Biol* **88**, 535-545 (1974).

5. Kobiler O, Rokney A, Friedman N, Court D, Stavans J, Oppenheim A. Quantitative kinetic analysis of the bacteriophage genetic network. *PNAS* **102**, 4470-4475 (2005).

6. Oppenheim AB, Kobiler O, Stavans J, Court DL, Adhya SL. Switches in Lambda Development. *Annu Rev Genet* **39**, 409-429 (2005).

7. Kourilsky P. Lysogenization by bacteriophage lambda-II. Identification of genes involved in the multiplicity dependent processes. *Biochimie* **56**, 1511-1515 (1974).

8. Sussman R, Jacob F. On a thermosensitive repression system in the Escherichia coli lambda bacteriophage. *CR Acad Sci* **254**, (1962).

9. Zhang N, Young R. Complementation and characterization of the nested Rz and Rz1 reading frames in the genome of bacteriophage lambda. *Mol Gen Genet* **262**, 659-667 (1999).

10. Babic A, Lindner AB, Vulic M, Stewart EJ, Radman M. Direct visualization of horizontal gene transfer. *Science* **319**, 1533-1536 (2008).

11. Szyf M*, et al.* DNA methylation pattern is determined by the intracellular level of the methylase. *Proceedings of the National Academy of Sciences of the United States of America* **81**, 3278-3282 (1984).

12. Sambrook J, and Russell, D.W. *Molecular Cloning: A Laboratory Manual*, Third edn. Cold Spring Harbor Press (2001).

13. Zeng L, Skinner SO, Zong C, Sippy J, Feiss M, Golding I. Decision making at a subcellular level determines the outcome of bacteriophage infection. *Cell* **141**, 682-691 (2010).

14. Yao NY, O'Donnell M. SnapShot: The replisome. *Cell* **141**, 1088, 1088 e1081 (2010).

15. Wegrzyn A, Wegrzyn G, Herman A, Taylor K. Protein inheritance: lambda plasmid replication perpetuated by the heritable replication complex. *Genes to cells : devoted to molecular & cellular mechanisms* **1**, 953-963 (1996).

16. Kelman Z, O'Donnell M. DNA polymerase III holoenzyme: structure and function of a chromosomal replicating machine. *Annual review of biochemistry* **64**, 171-200 (1995).

17. Maki H, Kornberg A. The polymerase subunit of DNA polymerase III of Escherichia coli. II. Purification of the alpha subunit, devoid of nuclease activities. *The Journal of biological chemistry* **260**, 12987-12992 (1985).

18. Gillespie DT. Approximate accelerated stochastic simulation of chemically reacting systems. *Journal of Chemical Physics*, 1716-1733 (2001).

19. Gillespie DT. Exact stochastic simulation of coupled chemical reactions. *Journal of Physical Chemistry*, 2340 – 2361 (1977).

20. Shao Q, Hawkins A, Zeng L. Phage DNA dynamics in cells with different fates. *Biophysical journal* **108**, 2048-2060 (2015).

21. Ueda K, McMacken R, Kornberg A. dnaB protein of Escherichia coli. Purification and role in the replication of phiX174 DNA. *The Journal of biological chemistry* **253**, 261-269 (1978).

22. Wu YH, Franden MA, Hawker JR, Jr., McHenry CS. Monoclonal antibodies specific for the alpha subunit of the Escherichia coli DNA polymerase III holoenzyme. *The Journal of biological chemistry* **259**, 12117-12122 (1984).
